# A retrospective review of genital fistula occurrence in nine African countries

**DOI:** 10.1186/s12884-022-05051-w

**Published:** 2022-10-04

**Authors:** Carrie J. Ngongo, Thomas J. I. P. Raassen, Marietta Mahendeka, Ladeisha Lombard, Jos van Roosmalen, Marleen Temmerman

**Affiliations:** 1 Global Health Division, RTI International, Research Triangle Park, USA; 2 Nairobi, Kenya; 3 Bugando Medical Centre, Mwanza, Tanzania; 4 Cape Town, South Africa; 5 Leiden University Medical Centre and Athena Institute VU University, Amsterdam, Netherlands; 6 Centre of Excellence in Women and Child Health, Aga Khan University, Nairobi, Kenya; 7 Faculty of Medicine and Health Sciences, Ghent University, Ghent, Belgium

**Keywords:** Cesarean section, Injury, Obstetric fistula, Surgery, Access, Quality of care

## Abstract

**Background:**

Female genital fistulas are abnormal communications that lead to urinary and/or fecal incontinence. This analysis compares the characteristics of women with fistulas to understand how countries differ from one another in the circumstances of genital fistula development.

**Methods:**

This retrospective records review evaluated demographics and circumstances of fistula development for 6,787 women who sought fistula treatment between 1994 and 2017 in Tanzania, Uganda, Kenya, Malawi, Rwanda, Somalia, South Sudan, Zambia, and Ethiopia.

**Results:**

Most women developed fistula during childbirth, whether vaginal (3,234/6,787, 47.6%) or by cesarean section (3,262/6,787, 48.1%). Others had fistulas attributable to gynecological surgery (215/6,787, 3.2%) or rare causes (76/6,787, 1.1%). Somalia, South Sudan, and Ethiopia had comparatively high proportions following vaginal birth and birth at home, where access to care was extremely difficult. Fistulas with live births were most common in Kenya, Malawi, Rwanda, Uganda, Tanzania, and Zambia, indicating more easily accessible care.

**Conclusions:**

Characteristics of women who develop genital fistula point to geographic differences in obstetric care. Access to care remains a clear challenge in South Sudan, Somalia, and Ethiopia. Higher proportions of fistula after cesarean birth and gynecological surgery in Kenya, Malawi, Rwanda, Uganda, Tanzania, and Zambia signal potential progress in obstetric fistula prevention while compelling attention to surgical safety and quality of care.

## Background

Female genital fistulas include vesico-vaginal fistulas, recto-vaginal fistulas, and ureteric injuries: abnormal communications between the genital tract and the bladder, urethra, ureters, colon, or rectum that lead to urinary and/or fecal incontinence. Most genital fistulas are obstetric, arising from pressure necrosis during prolonged, obstructed labor. Obstruction occurs when the presenting part of the fetus cannot progress into the birth canal despite strong uterine contractions, due to cephalo-pelvic disproportion, malpresentation, or malposition. Obstructed labor occurs in an estimated 4.6% of childbirths, causing 8% of maternal deaths [1]. Over 90% of women with obstetric fistula give birth to stillborn babies [2].

Obstetric fistula prevention requires appropriate labor monitoring and access to quality basic and comprehensive emergency obstetric care. High-income countries eliminated obstetric fistula by the mid-twentieth century [3]. These countries now report rare cases of genital fistula due to other causes, such as gynecological hysterectomy, radiotherapy, and cervical carcinoma [4, 5]. Iatrogenic fistula can occur as a complication of obstetric and gynecological surgery, especially cesarean section [6–8]. Genital fistula remains a challenge in low-resource settings where women do not have sufficient access to quality emergency obstetric care.

The relative rarity of genital fistula makes prevalence estimation difficult. Pooled prevalence from population-based studies indicates that one million women may have fistulas in sub-Saharan Africa and South Asia, with over 6,000 new cases per year [9]. Demographic and Health Survey estimates from 2005–2011 include urinary fistula symptoms point prevalence of 10.0 per 1,000 women of reproductive age in Uganda, 5.6 in Ethiopia, 5.0 in Kenya, 2.1 in Tanzania, and 1.5 in Malawi, which translates to urinary fistula symptoms in approximately 1 per 1000 women of reproductive age in sub-Saharan Africa [10]. The prevalence of fistula following gynecological surgery, trauma, and other causes is not known.

The objective of this paper is to compare the characteristics of women with fistula to understand how countries differ from one another in the circumstances of genital fistula development. Documenting geographic differences can help policymakers to tailor appropriate strategies to reduce fistula incidence.

## Methods

This retrospective records review evaluated the circumstances of fistula development for women who sought fistula treatment in nine African countries. Women were interviewed in 89 facilities in Tanzania, Uganda, Kenya, Malawi, Zambia, Rwanda, Ethiopia, Somalia, and South Sudan. Women consented to fistula repair, following each hospital’s counseling and informed consent process. The number of women in each country reflects the number of repairs conducted by the second and third author and colleagues in those countries. Data were collected between June 1994 and December 2017. We excluded women whose fistulas were of unclear origin or developed before 1980.

One of the surgeons interviewed each woman and recorded information on a standard form, documenting demographics, obstetric history, and duration of leaking [11]. Women who developed fistula during childbirth reported birth location and outcomes, including whether the baby lived more than two days (“alive”), died within two days (“early neonatal death”), or was stillborn. In case of multiples, births were counted “alive” if at least one baby was alive at birth. Most cesarean births in this series were emergency procedures; only five women had elective cesarean birth without labor. During fistula repair one of the surgeons noted the numbers of fistula and type(s), using the Waaldijk classification system [12]. The unit of analysis in Table 3 was fistulas; elsewhere the unit of analysis was women.

Data were entered into an Excel database, with names changed to unique identification numbers to protect women’s privacy. Data were analyzed using Stata 16 software (StataCorp).

## Results

This review includes 6,787 women who sought treatment for genital fistula. Table 1 provides characteristics of the women with fistula by country. Most variables are reported for all women seeking genital fistula repair, including fistula number and type, age at fistula development, education, duration of leaking, maternal height, and relationship status. Previous abdominal surgery, place of birth, and stillbirth were applicable only to women who developed fistula during childbirth, whether vaginal or by cesarean birth.

**Table 1:** Demographics of women with genital fistula

	Tanzania		Uganda		Kenya		Malawi		Zambia		Rwanda		Ethiopia		Somalia		South Sudan		Total	
Women	2213		1527		1131		662		168		418		110		316		242		6787	
Fistulas	2439		1655		1233		703		182		456		134		345		281		7428	
Incontinence																				
Both urine and feces	185	8.4%	98	6.4%	79	7.0%	31	4.7%	5	3.0%	23	5.5%	30	27.3%	28	8.9%	40	16.5%	519	7.6%
Urine	1996	90.2%	1368	89.6%	1006	88.9%	609	92.0%	153	91.1%	382	91.4%	74	67.3%	258	81.6%	177	73.1%	6,023	88.7%
Feces	32	1.4%	61	4.0%	46	4.1%	22	2.0%	10	6.0%	13	3.1%	6	5.5%	30	9.5%	25	10.3%	245	3.6%
Place of birth																				
Home	209	9.7%	159	10.7%	202	18.4%	66	10.2%	10	6.1%	42	10.1%	37	35.6%	96	31.4%	93	38.8%	914	13.8%
Health facility	1941	90.3%	1329	89.3%	893	81.6%	579	89.8%	153	93.9%	374	89.9%	67	64.4%	210	68.6%	147	61.3%	5693	86.2%
Baby condition																				
Stillbirth	1777	84.0%	1106	75.7%	829	77.8%	485	77.1%	120	74.5%	347	84.4%	94	92.2%	257	84.3%	201	84.1%	5216	80.4%
Live birth	260	12.3%	258	17.7%	174	16.3%	122	19.4%	31	19.3%	57	13.9%	7	6.9%	38	12.5%	32	13.4%	979	15.1%
Early neonatal death	79	3.7%	97	6.6%	63	5.9%	22	3.5%	10	6.2%	7	1.7%	1	1.0%	10	3.3%	6	2.5%	295	4.5%
Age at fistula development																				
<19	778	35.2%	597	39.1%	461	40.8%	200	30.2%	62	36.9%	83	19.9%	42	38.2%	137	43.4%	100	41.3%	2460	36.2%
20-29	916	41.4%	599	39.2%	423	37.4%	256	38.7%	67	39.9%	207	49.5%	44	40.0%	118	37.3%	109	45.0%	2739	40.4%
>30	519	23.5%	331	21.7%	247	21.8%	206	31.1%	39	23.2%	128	30.6%	24	21.8%	61	19.3%	33	13.6%	1588	23.4%
Education																				
None	767	35.0%	577	38.6%	305	28.2%	219	33.1%	70	41.7%	137	32.8%	80	76.2%	270	86.0%	190	78.8%	2615	39.2%
Primary incomplete	1373	62.6%	878	58.8%	504	46.5%	373	56.4%	76	45.2%	254	60.8%	20	19.0%	26	8.3%	48	19.9%	3552	53.2%
Primary complete	54	2.5%	39	2.6%	274	25.3%	69	10.4%	22	13.1%	27	6.5%	5	4.8%	18	5.7%	3	1.2%	511	7.7%
Duration of leaking																				
<6 months	745	33.7%	408	26.9%	343	30.5%	119	18.4%	54	32.1%	42	10.1%	30	27.8%	58	18.4%	50	20.7%	1849	27.4%
6-12 months	387	17.5%	242	28.9%	188	16.7%	92	14.3%	12	7.1%	22	5.3%	18	16.7%	76	24.1%	38	15.7%	1075	15.9%
1-5 years	591	26.8%	439	28.9%	357	31.8%	165	25.6%	35	20.8%	98	23.5%	30	27.8%	114	36.1%	94	38.8%	1923	28.5%
5-10 years	263	11.9%	220	14.5%	134	11.9%	121	18.8%	21	12.5%	129	30.9%	15	13.9%	44	13.9%	34	14.0%	981	14.5%
>10 years	222	10.1%	209	13.8%	102	9.1%	148	22.9%	46	27.4%	126	30.2%	15	13.9%	24	7.6%	26	10.7%	918	13.6%
Relationship status at time of repair																				
Living with husband	1,151	52.2%	765	50.3%	546	48.9%	408	61.6%	115	68.5%	218	52.2%	55	50.9%	157	49.7%	132	54.5%	3547	52.5%
Living with family	386	17.5%	304	20.0%	114	10.2%	159	24.0%	34	20.2%	125	29.9%	40	37.0%	52	16.5%	70	28.9%	1284	19.0%
Living alone	409	18.5%	319	21.0%	212	19.0%	55	8.3%	9	5.4%	57	13.6%	10	9.3%	97	30.7%	29	12.0%	1197	17.7%
Remarried	50	2.3%	29	1.9%	14	1.3%	38	5.7%	9	5.4%	13	3.1%	-	0.0%	4	1.3%	5	2.1%	162	2.4%
Never married	183	8.3%	61	4.0%	216	19.3%	-	0.0%	1	0.6%	3	0.7%	3	2.8%	1	0.3%	4	1.7%	472	7.0%
Widowed	27	1.2%	43	2.8%	15	1.3%	2	0.3%	-	0.0%	2	0.5%	-	0.0%	5	1.6%	2	0.8%	96	1.4%
Previous abdominal surgery	166	7.6%	156	10.3%	134	12.0%	60	9.1%	15	9.0%	54	12.9%	3	2.9%	14	4.5%	11	4.6%	613	9.1%
Height (median, IQR)	151	147-155	154	150-158	154	150-158	150	146-155	152	147-157	151	147-155	152	148-159	155	150-160	160	155-164	153	148-157

Most women had a single fistula (5,738, 84.5%), while 1,049 (15.5%) presented with multiple fistulas, meaning a combination of fistulas in different locations. Ethiopia had the highest proportion of women incontinent of both urine and feces due to the combination of vesico- and recto-vaginal fistulas (30/110, 27.3%), followed by South Sudan (40/242, 16.5%) (Table 1).

More than 40% of women developed fistula during adolescence in Somalia (137/316, 43.4%), South Sudan (100/242, 41.3%), and Kenya (461/1,131, 40.8%), while the proportion of women developing fistula after age 30 was highest in Malawi (206/662, 31.1%) and Rwanda (128/418, 30.6%). More than one quarter of women with fistula had been incontinent for ten years or longer in Rwanda (126/417, 30.2%) and Zambia (46/168, 27.4%).

Most women with fistula had not completed primary school, and 39.2% (2,615/6,678) had not attended formal school at all. Low educational access was particularly pronounced in Somalia (270/314, 86.0%), South Sudan (190/241, 78.8%), and Ethiopia (80/105, 76.2%).

Women who developed fistula during labor and childbirth most commonly reported giving birth in health facilities (5,693/6,607, 86.2%), with proportions near or above 90% in Zambia (153/163, 93.9%), Tanzania (1,941/2,150, 90.3%), Rwanda (374/416, 89.9%), and Uganda (1,329/1,488, 89.3%). In contrast, between 60 and 70% of women with fistula had given birth in health facilities in South Sudan (147/240, 61.3%), Ethiopia (67/104, 64.4%), and Somalia (210/306, 68.6%). The proportion of women with fistula who gave birth to a live baby varied by country. While 5,216/6,490 (80.4%) women reported a stillbirth overall, the percentage varied from 94/102 (92.2%) in Ethiopia to 120/161 (74.5%) in Zambia (Table 1).

Table 2 groups women according to the circumstances of their fistula development, whether vaginal birth, cesarean birth, gynecological surgery, or other causes. “Cesarean birth” includes cesarean section, cesarean hysterectomy, and uterine rupture repair. “Other causes” include trauma (from accidents, traditional healers, or sexual violence), congenital fistula, and fistula related to abortion, HIV infection, or radiation, as further explored elsewhere [13].

**Table 2:** Circumstantial origin of women's genital fistulas, by country

Circumstantial origin of genital fistula	Tanzania		Uganda		Kenya		Malawi		Zambia		Rwanda		Ethiopia		Somalia		South Sudan		Total	
Vaginal birth	1047	47.3%	620	40.6%	520	46.0%	291	44.0%	73	43.5%	204	48.8%	87	79.1%	232	73.4%	160	66.1%	3234	47.6%
Cesarean birth	1069	48.3%	844	55.3%	552	48.8%	341	51.5%	88	52.4%	202	48.3%	16	14.5%	73	23.1%	77	31.8%	3262	48.1%
Gynecological surgery	78	3.5%	52	3.4%	41	3.6%	22	3.3%	5	3.0%	10	2.4%	2	1.8%	2	0.6%	3	1.2%	215	3.2%
Other	19	0.9%	11	0.7%	18	1.6%	8	1.2%	2	1.2%	2	0.5%	5	4.5%	9	2.8%	2	0.8%	76	1.1%
**Total**	**2213**		**1527**		**1131**		**662**		**168**		** 418**		** 110**		** 316**		**242**		** 6787**	

Most genital fistulas followed childbirth, whether vaginal (3,234/6,786, 47.7%) or by cesarean section (3,262/6,787, 48.1%) (Table 2). Fistula following cesarean birth was most common in women in Uganda (844/1,527, 55.3%), Zambia (88/168, 52.4%), and Malawi (341/662, 51.5%). In contrast, high proportions of fistula followed vaginal births in Ethiopia (87/110, 79.1%), Somalia (232/316, 73.4%), and South Sudan (160/242, 66.1%) (Table 2).

Just over 3% of women developed fistula from gynecological surgery (215/6,787, 3.2%), while 1.1% of women developed fistula through other circumstances (76/6,787). The proportion of fistulas following gynecological surgery was highest in Kenya (41/1,131, 3.6%), Tanzania (78/2,213, 3.5%), Uganda (52/1,527, 3.4%) and Malawi (22/662, 3.3%), while fistulas following gynecological surgery constituted less than 2% of fistulas repaired in Somalia, South Sudan, and Ethiopia (Table 2).

Fistula types differ by circumstances of development. Iatrogenic ureteric injuries and vault fistulas occur after cesarean birth or gynecological surgery (Table 3) [11, 14]. The exceptions were “postrepair” ureteric injuries that arose during fistula surgery in women who had initially developed other fistulas during childbirth. Over one quarter of fistulas following cesarean birth were vesico-[utero]/-cervico-vaginal (26.1%, 914/3,508), compared to just 6.8% (244/3,614) of fistulas following vaginal birth.

**Table 3:** Genital fistula type by circumstantial origin

Fistula Type		Vaginal birth		Cesarean birth		Gynecological surgery	
Vesico-[utero]/-cervico-vaginal (Type I)		244	6.8%	914	26.1%	4	1.8%
Vault (Type I)		-	-	131	3.7%	129	58.1%
Ureteric (Type III)		30	0.8%	279	8.0%	70	31.5%
Involving closing mechanism:							
Type II Aa (without (sub)total urethra involvement, without circumferential defect)		1615	44.7%	1484	42.3%	5	2.3%
Type II Ab (without (sub)total urethra involvement, with circumferential defect)		464	12.8%	268	7.6%	-	-
Type Ba (with (sub)total urethra involvement, without circumferential defect)		343	9.5%	162	4.6%	7	3.2%
Type Bb (with (sub)total urethra involvement, with circumferential defect)		220	6.1%	65	1.9%	-	-
Recto-vaginal fistula		576	15.9%	130	3.7%	5	2.3%
Irreparable fistula		17	0.5%	10	0.3%	1	0.5%
Other (urethral incontinence, bladder stone, vaginal stenosis)		105	2.9%	65	1.9%	1	0.5%
	**Total**	**3614**		**3508**		**222**	

Previous abdominal surgery complicates cesarean section. Nearly all reported previous abdominal operations were cesarean sections (98.5%, 601/610). Others included two bilateral tubal ligations, two myomectomies, one myomectomy and salpingectomy, and four other laparotomies (for intestinal obstruction, abortion, ectopic pregnancy, or menorrhagia). In this series over 10% of women with fistula after childbirth reported previous abdominal surgery in Rwanda (54/417, 12.9%), Kenya (134/1,116, 12.0%), and Uganda (156/1,518, 10.3%) (Table 1). While just 4.1% (132/3,229) of women developing fistula after vaginal birth reported previous abdominal surgery, the frequency was much higher for those developing fistula after cesarean section (13.4%, 341/2,554), cesarean section/hysterectomy (13.9%, 83/598), uterine rupture repair (18.7%, 20/107), and gynecological surgery (16.3%, 34/209) (Table 4).

**Table 4:** Previous abdominal operation by circumstantial origin of genital fistula

	Previous abdominal operation				
Circumstantial origin of genital fistula	No	%	Yes	%	Total
Vaginal birth	3097	95.9%	132	4.1%	3229
Cesarean birth					
Cesarean section	2213	86.7%	341	13.4%	2554
Cesarean section/hysterectomy	515	86.1%	83	13.9%	598
Uterine rupture repair	87	81.3%	20	18.7%	107
Gynecological surgery	175	83.7%	34	16.3%	209

**Fig. 1:**
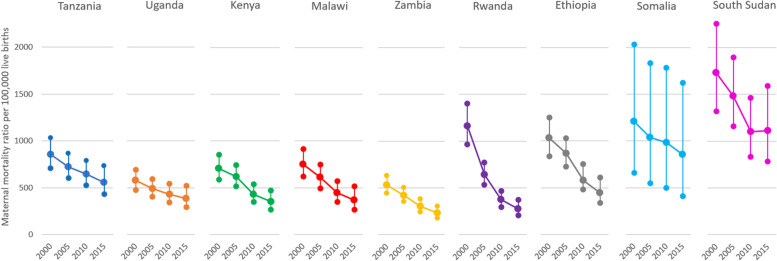
Country differences in maternal mortality ratios (World Health Organization)

Country differences are evident in World Health Organization data on maternal deaths per 100,000 live births between 2000 and 2015 (Fig. 1). All countries have made progress in reducing maternal mortality ratios (MMRs) over the period, but with differing starting points and speed. MMRs in Somalia and South Sudan in 2015 were higher than in most other countries in 2000. Change was most rapid in Rwanda and Ethiopia, where MMRs fell from more than 1000 per 100,000 live births in 2000 to fewer than 500 by 2015.

**Fig. 2:**
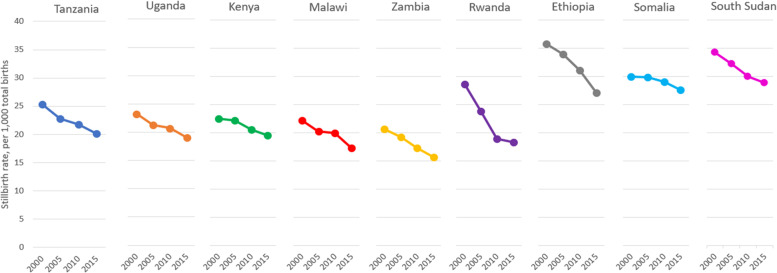
Country differences in stillbirth rate (World Health Organization)

Stillbirth rates from the World Health Organization similarly give insight into country differences in care during childbirth (Fig. 2). All countries made progress in reducing stillbirth rates between 2000 and 2015, with Rwanda and Ethiopia experiencing the most dramatic change. Progress was most muted in Somalia. Stillbirth rates were highest in Ethiopia, South Sudan, and Somalia. Stillbirth rates were lowest in Zambia and Malawi.

## Discussion

Policymakers and advocates have long emphasized access to care: women experiencing obstetric complications should be able to reach health facilities offering basic and comprehensive emergency obstetric care. This comparison reveals important country-level differences in the characteristics of women who develop genital fistula. The emerging story is not simple lack of access to health care, but of both lack of access and quality of care.

Some women experiencing prolonged, obstructed labor give birth at home without accessing a health facility, indicating comparative challenges in access to care. This was most common in South Sudan, Somalia, and Ethiopia, where a substantial share of genital fistulas followed childbirth at home. Women who developed fistula after birth at home are survivors; others in their circumstances might have died. The complexity of such cases is reflected in the high proportion of women with multiple fistulas who experience both urinary and fecal incontinence. The proportions of home birth and multiple fistulas reported by women with fistula in South Sudan, Somalia, and Ethiopia correspond to high MMRs and high stillbirth rates illustrated by World Health Organization data in Figs. 1 and 2.

Most women with genital fistula gave birth in health facilities. Although some women arrive late in health facilities, one report found that nearly three-quarters of women with fistula presented to hospitals or health centers during early labor [15]. Appropriate labor monitoring, decision-making, and referrals are crucial. The proportion of facility births and specifically cesarean births among women with fistula indicates greater comparative access to obstetric care in Kenya, Malawi, Rwanda, Tanzania, Uganda, and Zambia as compared to Ethiopia, South Sudan, and Somalia.

Cesarean section can be a lifesaving procedure for women experiencing prolonged, obstructed labor. Cesarean birth is increasingly common, both generally at population level and specifically among women who later present for fistula repair [16, 17]. Some of this increase is saving lives and preventing fistula, a positive outcome that is not captured in this population of women who developed fistula.

Despite its important place in enabling fistula elimination, however, rising numbers of cesarean births increase the risk of all associated complications, including iatrogenic fistula. Location and circumstances can indicate that a fistula is iatrogenic rather than attributable to pressure necrosis [7, 11]. Evidence has been mounting about the proportion of iatrogenic fistula amongst repaired fistulas [18–21]. In a separate analysis we reported that 26.8% (787/2,942) of women with fistula after cesarean section have injuries caused by surgery rather than pressure necrosis from prolonged, obstructed labor [7]. This aligns with reports from others [6]. Iatrogenic vesico-[utero]/-cervico-vaginal, vault, and ureteric fistulas generally occur after cesarean birth and gynecological surgery, not after vaginal birth. In this series 80% of women with fistula following childbirth had stillbirths, in contrast to 90% reported by other investigators [2]. Together, these observations support a conclusion that some of these fistulas following childbirth are the result of surgical complications rather than unattended prolonged, obstructed labor.

One interpretation of these data is that countries are at different points on their developmental journeys toward fistula elimination. In South Sudan, Somalia, and Ethiopia, access to emergency obstetric care remains difficult. Widespread insecurity and danger in South Sudan and Somalia have created particular challenges for women experiencing obstetric complications. Ethiopia’s population is largely rural and spread across difficult terrain.

In contrast, Kenya, Malawi, Rwanda, Uganda, Tanzania, and Zambia are likely further along on the journey toward reducing the incidence of obstetric fistula, witnessing higher proportions of fistula following cesarean birth and gynecological surgery (Table 2). Given the risk of iatrogenic fistula following cesarean birth, a less positive interpretation could be that these countries are providing increasing numbers of cesarean births without ensuring that surgeries are safely performed following the right indications. As women have greater access to health care, emphasis must be placed on its quality.

### Strengths and limitations

The strength of this study is the size and geographic breadth of its sample, which offers a unique opportunity to assess the circumstances of fistula development in nine African countries. We documented women’s demographic information and circumstances of fistula development in detail. The study is not without limitations, however. Included countries have different sample sizes, reflecting variation in where the second and third author and colleagues repaired fistulas. While women sought repair in multiple hospitals with wide catchment areas in most countries, the countries with large sample sizes may be more representative of all women seeking fistula repair than those with smaller samples. This series includes women who sought surgical treatment for their fistula. It cannot include women who developed fistula but did not reach treatment centers. Overall fistula circumstances may be different among all women with fistula if particular women are more likely than others to reach treatment centers. Demographic information and data on birth experiences relied on women’s accounts of past events. Women know their obstetric history well, but in some cases many years passed between the original event and fistula treatment. We acknowledge that women’s recollection of childbirth may differ from how providers diagnose obstetric complications. We did not have medical records information on the duration of labor or indications for cesarean birth (where applicable). Additional research is needed on surgical complications from cesarean birth.

## Conclusions

Continued progress toward fistula elimination requires countries to ensure adequate infrastructure and security for women to access basic and comprehensive emergency obstetric care. Providers should have the knowledge, experience, and environment to be able to provide quality care to all women, particularly those who experience labor complications. Facilities and providers should consider the quality of emergency obstetric care, including timing and decision-making for cesarean birth. Safe surgical care requires robust provider training, supervision, and mentoring, appropriate work environments, and clear safety standards. Fistula elimination will require a holistic systems approach that improves emergency obstetric care and surgical safety.

## Data Availability

Data generated and analysed during the current study are not publicly available during a period of analysis and dissemination but will be available from the corresponding author on reasonable request.
